# Notes of the Washington Congress

**Published:** 1887-10

**Authors:** R. B. White

**Affiliations:** Ennis, Texas


DANIEL'S
### TEXAS MEDICAL JOURNAL.

PUBLISHED MONTHLY.—SUBSCRIPTION $2.00 A YEAR.

Vol. 3. AUSTIN, OCTOBER, 1887. No. 4.

### ORIGINAL ARTICLES.

☞CONTRIBUTED EXCLUSIVELY TO THIS JOURNAL.☜

*The Articles in this Department are accepted and published with the understanding that we are not responsible for, nor do we indorse the views and opinions of the writers by so doing.*

### NOTES OF THE WASHINGTON CONGRESS.

*By R. B. White, M. D., Ennis, Texas.*

For Daniel's Texas Medical Journal.

TO the great majority of even well informed Europeans, the sarcastic query of Sidney Smith, "Who reads an American book?' still implies a world of meaning. Among the events that are fast erasing that ancient stigma, a foremost place must be assigned to the Ninth International Medical Congress. Never before in the history of America has there been such an opportunity for a comparison of the intellectual status of the citizens of the Republic with their compeers in other lands, and when the records of the Congress are given to the world, it will be apparent, to even the most prejudiced, that the medical profession in America is fully of age.

The opening scene of the Congress was a sight never to be forgotten; the entire space of Albaugh's large Opera House was crowded; at least three thousand physicians were present, besides large numbers of the elite of both sexes of the National Capital. Competent judges estimated the throng at fully five thousand persons. Many who had been invited to witness the initial exercises were unable to obtain places, owing to the great crowd.

Promptly at eleven o'clock a. m., on Monday, September 5, the curtain rose, and the assembled audience beheld a truly rare sight. The central figure on the stage was President Cleveland, with Secretary Bayard and Speaker Carlisle on either hand; a little removed the well known figure of Dr. N. S. Davis, of Chicago, was recognized; with him were Dr. John B. Hamilton, Secretary General of the Congress, Dr. Garnett, Dr. Smith, of Philadelphia, Dr. J. M. Toner, Dr. W. B. Atkinson, Secretary American Medical Association, and several other prominent American physicians and active promoters of the Congress. Representative physicians from nearly every foreign country occupied places on the stage. England was represented by Drs. Pavy, Graily Hewitt and Phillips, of London, and by Inspector General Lloyd, of the British Navy. Drs. Unna and Martin represented Germany. From France came the famous Dr. Leon Le-Fort, Prof. Semmola, of Italy; Dr. Rohier, of St Petersburg, and Dr. Alvarado, of Mexico, represented their respective countries.

The doctors showed their appreciation of the presence of the President of the United States, by enthusiastic and well sustained applause. Quiet being restored, President Cleveland formally opened the Congress, and business was rapidly despatched.

When it was known that the Ninth International Medical Congress would meet in America, it was almost a matter of course that the late Dr. Austin Flint should be its chief officer. His nomination to the office of President by the Executive Committee was felt to be peculiarly appropriate, and his lamented death, at a time when his great influence was being exerted to restore harmony in the divided councils of the Congress, was especially unfortunate. However, the appointment of Dr. Davis to the vacant place restored the confidence of the profession to a great extent. He who had labored so industriously, and with such great success in organizing the profession in America, would surely fill the highest office in the greatest organization of the medical men of the world in a becoming manner; his election as President by acclamation was a fitting tribute to the sterling qualities of Dr. Davis, and to the untiring energy he had displayed in overcoming numerous obstacles to the success of the Congress.

The selection of Dr. John B. Hamilton for the important office of Secretary General of the Congress, was a deserved recognition of the great industry and good judgment displayed by that gentleman in the preliminary work of the Congress. His duties have been peculiarly arduous, and often of a delicate nature, but always performed in such a manner as to defy adverse criticism. In giving a detailed history of the origin and organization of the Congress, he was necessarily compelled to notice the defection of the original committee, after the meeting of the American Medical Association, in New Orleans, 1885; but this was done with so much tact that no one would have suspected the least want of harmony in the preliminary organization; the mal-contents will derive little pleasure from Dr. Hamilton's report.

The principal feature of the general morning sessions was an address by some one of world-wide fame, that of President Davis coming first on Monday. Dr. Davis' remarks might well serve as a model for those who will occupy similar places in future medical gatherings. He touched on many of the great medical problems of the day, and called special attention to the collective investigation of disease, urging the necessity for extending and systematising the agencies already in operation for that purpose.

On Tuesday the morning hour was occupied by Prof. Austin Flint, who made a masterly synopsis of all that is known of the causation, mechanism and treatment of fever. He paid a due tribute to Chassat and Graves, the pioneers in the supportive plan of treatment, that has now become quite a matter of routine. Among antipyretics, he gave the preference to antipyrin, as, in addition to its wonderful efficacy in lowering temperature, it has the property of diminishing the excretion of nitrogen. Dr. Flint's address recalled, in the most marked manner, the teaching of his late distinguished father; especially in his high appreciation of alcohol, both as a refrigerant and as an aliment, one was reminded of the positive stand taken by the elder Flint on this important topic.

On Wednesday Dr. Unna, of Hamburg, gave an address on Dermatology, in German, that was much praised by those competent to pass on it.

On Thursday the address was by Prof. Semmola, of Naples, Italy, and was the most disappointing of any delivered in the general sessions; perhaps too much had been expected of him. Dr. Durante was a delegate to the Congress from the Italian government, and the Executive Committee had requested him to reply on behalf of Italy to the address of welcome at the opening of the Congress, and the Doctor had accepted; this happened months before the time of meeting. Later Prof. Semmola was also made a delegate by the Italian government; and he, being a senator, also, claimed as a right, the position that had already been assigned to Prof. Durante. Prof. Semmola was also to read a paper at a general session, in which it was announced that he would report the result of some highly important work in bacteriology, and would take direct issue with Koch in certain of his conclusions. It was understood that unless Prof. Semmola was allowed to make the reply for Italy, he would withdraw from the Congress, and of course his paper would not be presented; so, to prevent this result, and for other reasons, the committee gave Prof. Semmola the position already assigned Dr. Durante. The paper, far from containing original matter, proved quite a commonplace affair; its reference to Koch and his doctrines consisting in some general statements to the effect that the germ theory could not prove a basis for scientific therapeutics, as any remedy that would destroy the life of the microbes would be fatal also to the human organism; a proposition which is surely not universally true. The whole Durante-Semmola affair was rather unfortunate, as after Dr. Durante had been superseded by Prof. Semmola, the former gentleman withdrew from the Congress in high dudgeon, and, though afterwards induced to return, still many thought he had been rather hardly used; it was, however, about the only inharmonious incident of the meeting, but it was pounced on by the daily papers, and became the subject of considerable and conflicting comment.

A very interesting and important paper by Dr. Blandford, of London, was read on Friday morning, and concluded the series of the general addresses. Dr. Blandford has long been recognised as an able and original worker in psychology, and on this occasion directed his remarks to the treatment of certain forms of insanity. He related the histories of a number of cases of mental trouble coming under his own care, and showed that the residence in an asylum for even the shortest period, might be the means of blasting the future lives and prospects of a large number of worthy people of both sexes, while the same individuals would not suffer in reputation or in their own estimation by being secluded for a time either in their own homes or at the sea-side. From his own extended observation, the speaker related the history of quite a large number of cases, some of which had been subject to asylum treatment from the very inception of their malady, while others were treated at their own private residences, or perhaps at a quiet watering place. The results certainly seemed to prove that in properly selected cases, recovery is even more likely to be speedy under the latter plan, while saving the patient from the mortification and stigma that unavoidably adheres to a residence in an asylum.

Dr. Blandford laid down plain and comprehensive rules by which the general practitioner can usually distinguish the cases in which asylum treatment is a necessity, from those which may safely be cured outside. Certainly no subject can be of more importance, and Dr. Blandford's paper will well repay careful study, and it is likely to be the means of calling more general attention to a matter of vital interest to those unfortunates who are temporarily bereft of reason. The general country practitioner gives the diagnosis and treatment of insanity far too little consideration, and there is no doubt that if the subject was better understood, especially the capabilities of home treatment in the early stages, which come under the observation of the family physician, rather than the specialist, many poor sufferers, who are now dragging out a miserable life within the walls of our asylums, might be saved to fill positions of usefulness and honor.

The Ninth International Medical Congress was, above all things, a convention of workers; the great majority of its members were on the ground one to three days prior to the opening. When the various sections were called to order at 3 o'clock p. m. on Monday, the quiet business-like air of the different presidents and secretaries gave assurance that the working affairs of the Congress were in competent hands.

The places of meeting of the several sections were as nearly central as could be expected, and when it is remembered that provisions had to be made for eighteen separate rooms, the majority of which had to be large halls, it will be apparent that it was no slight task to provide the elegant and spacious apartments in which the sections held their sessions.

The great variety of work, including almost every topic in medicine, surgery, and the specialties, that was indicated by the printed programme, enabled the members to attend any section at the time the subjects in which they were especially interested were being considered. It was really surprising how little time was wasted in passing from one section to another. Washington City has an excellent street railway service, and a fine system of Herdic lines; then, for the very modest sum of twenty-five cents one could hire a cab for thirty to sixty minutes; by using one or other of these modes of conveyance, the rounds of all the sections could be made in a few minutes.

The subject of general medicine ought to be of vastly more importance to physicians than any other branch of the science, as the vast majority of the diseases they are called on to treat, come under that head; yet the section devoted to general medicine at Washington was but poorly attended, and its programme showed a decided paucity in papers contributed. The matter was noticed and commented on, and various explanations given; one eminent authority held that the best talent in the profession is being absorbed by the specialties, a result which, if true, must in time react disastrously on both general medicine and the special branches.

A very important paper contributed to this section, was by Dr. Ephraim Cutter, of New York, entitled "The Morphology of Rheumatic Blood." The author sought to show that in rheumatism the blood, when examined microscopically, invariably displays concretions of oxalate of lime, cystine, phosphatic and uric acid, constituting, as it were, a gravel of the blood; that the treatment proper for any given case could always be determined by examining a drop of the patient's blood, and ascertaining what particular variety of concretion was present; such an examination, too, would show when treatment might be safely discontinued. Dr. Cutter's discoveries throw much needed light on the connection of heart disease with rheumatism. The granules of oxalate of lime, etc., present in the blood being deposited on the valves, pericardium, and other structures of the central organ of the circulation, lights up an active and often fatal inflammation. As the remedy suitable to any given case would depend on the kind of granule existing in the blood, this theory explains why in one case our treatment is eminently successful, while in another case, though the clinical features might be exactly the same, our remedies totally failed; owing to the fact that in one case the irritant was, say, oxalate of lime, while in the other uric acid was present; to remove which a totally different set of drugs was needed.

Dr. Cutter's paper was illustrated by some beautiful colored micro-photographs, in which the foreign bodies in the blood referred to were well shown on the screen, (the hall being first properly darkened); by means of a very fine stereopticon. This mode of illustration, by the stereopticon, was used in suitable instances in nearly every section, constituting a pleasing break in the monotony of ordinary discussion; but it is to be feared that many papers that were well received and highly instructive when read with these aids, will be barely intelligible when published in the ordinary way as the Transactions, and this is really a serious objection to this mode of illustrating the work of a scientific Congress.

The question of the proper treatment for phthisis was brought up in connection with papers read in this (General Medicine) and other sections, and it is worthy of note that whenever Bergeon's treatment by rectal injection was spoken of, it was mentioned only to be condemned as useless. A like verdict is to be inferred for pneumatic differentiation by means of the air-tight cabinet, as nothing was heard of that lately much vaunted plan, though a number of gentlemen were present who have had a good deal of experience with the pneumatic cabinet.

It is generally admitted that American physicians and surgeons, while frequently pioneers in new methods of treatment and original surgical devices, are not distinguished for experimental work in the abstract, such as sometimes forms the life-long employment of their European confreres. There have been, however, some notable exceptions; every one will recall the case of Dr. Beaumont, of St. Louis, who, many years ago, in a frontier town of the then wild West, made and recorded the results of a series of experiments on the functions of the stomach that still do duty in most of our standard text books of physiology. That the establishment of suitable laboratories for this kind of experimental work in Amer-ca will yield a rich fruitage, is shown by some of the reports made to the Washington Congress. A very interesting case in point was a paper read in the Section of Pathology, by Dr. E. O. Shakspeare, of Philadelphia, in which the author gave the particulars of a series of experiments made on rabbits to test the inoculability of tetanus. Portions of the medulla spinalis from an infected animal were injected beneath the dura-mater of the animals experimented on; the results of a large number of such inoculations were entirely uniform; by drying (in a certain manner) the morbid material before being injected the resulting symptoms were much modified. Dr. Shakspeare did not formulate any definite conclusions, as he is still pursuing his studies in inoculation, the results of which will be given to the profession in due time. These experiments of Dr. Shakspeare are calculated to throw much light on the Pasteur treatment for hydrophobia.

Another American worker who contributed original work to the Section of Pathology, was Dr. Victor Vaughn, of the University of Michigan. The Doctor has succeeded in isolating pyrotoxicon, a ptomaine peculiar to milk and its products. In another paper read in the Section of Diseases of Children, Dr. Vaughn pointed out the relation that this peculiar poison bears to the diseases of infancy, especially in children raised by hand.

Dr. Whitmarsh, of London, in a peculiarly well written paper, reviewed the subject of inoculation as a preventive of rabies. According to Dr. Whitmarsh, Pasteur is entirely a fanatic on the subject of hydrophobia, and his methods for the prevention of that disease are vitiated by a want of scientific exactitude; that, at the most, Pasteur can only be ranked as a successful empiric.

When Dr. Whitmarsh's paper was read (which, in addition to its slight appreciation of Pasteur's work, contained some of the stock arguments against vaccination) there was no one present in the section who felt either able or disposed to champion the views of the celebrated Frenchman; indeed, from the fact that the section awarded a special vote of thanks to the author of the paper, it might be inferred that the Congress had condemned Pasteur's treatment; there is good reason to believe that the reverse would be nearer the truth, for, on the evening following the discussion just referred to, the question was the topic of an informal conversation in the rotunda of the Riggs House. Here certain American members interested in Pasteur's plan, but who were not present in the Section of General Medicine when Dr. Whitmarsh's paper was read, expressed their convictions that Pasteur had made a great discovery; and, while there might be some technical details still needing to be perfected, the principle was sound. This was the opinion of gentlemen who had visited Pasteur's laboratory in Paris, and who had seen for themselves the practical working of the method. Reference was also made to the commission of experts, who had been sent to France by the British Government, and who, after thoroughly examining Pasteur's work, made a report entirely favorable; this report, among other things, contained an analysis of all the statistics available on the subject, which, more than anything else, showed the marked saving of life that had resulted from Pasteur's inoculations. Several distinguished Frenchmen, among whom were Dr. Apostoli and Dr. Pouisser, were interrogated as to the opinion held in France of the new treatment for rabies; much diversity was shown in their replies, but it was evident that while Pasteur has been opposed and almost denounced by some of the very highest medical authorities in his own country, yet the great bulk of the profession there have confidence in him, and believe that his efforts will be crowned with success. Dr. Pouisser remembered well with what incredulity people heard the announcement that chicken cholera and authrax in the domestic animals could be prevented by inoculation; but now such inoculation had become a matter of course over large districts in Europe, and anthrax, especially in sheep, had been almost stamped out. The recollection of instances of this kind is certainly a wonderful stimulus to faith in Pasteur's genius.

It is to be remembered that the foregoing notes are no sort of an index to the work done at Washington, but are simply a brief and imperfect synopsis of what came under the writer's individual observation in the departments referred to. Some account of the sections devoted to gynæcology and general surgery will be given at another time.

